# Genetic Barrier to Direct Acting Antivirals in HCV Sequences Deposited in the European Databank

**DOI:** 10.1371/journal.pone.0159924

**Published:** 2016-08-09

**Authors:** Dimas Alexandre Kliemann, Cristiane Valle Tovo, Ana Beatriz Gorini da Veiga, André Luiz Machado, John West

**Affiliations:** 1 Graduate Program in Medicine, Hepatology, Universidade Federal de Ciências da Saúde de Porto Alegre (UFCSPA), Porto Alegre, RS, Brazil; 2 Medical Infectologist, Hospital Nossa Senhora da Conceição (HNSC), Porto Alegre, RS, Brazil; 3 Department of Basic Health Sciences, Laboratory of Molecular Biology, Graduate Program in Pathology, Universidade Federal de Ciências da Saúde de Porto Alegre (UFCSPA), Brazil; 4 School of Biological Sciences, Nebraska Center for Virology, University of Nebraska, Lincoln, Nebraska, United States of America, University of Nebraska, Lincoln, NE, United States of America; University of North Carolina at Chapel Hill School of Dentistry, UNITED STATES

## Abstract

**Background & Aims:**

Development of resistance results from mutations in the viral genome, and the presence of selective drug pressure leads to the emergence of a resistant virus population. The aim of this study was to analyze the impact of genetic variability on the genetic barrier to drug resistance to DAAs.

**Methods:**

The genetic barrier was quantified based on the number and type of nucleotide mutations required to impart resistance, considering full-length HCV NS3, NS5A and NS5B regions segregated by genotype into subtypes 1a, 1b, 2a, 2b and 3a. This study analyzeds 789 NS3 sequences, 708 sequences and 536 NS5B sequences deposited in the European Hepatitis C Virus Database, in the following resistance-associated positions: NS3: F43/I/L/S/V, Q80K/R, R155K/G, A156G/S/T and D168A/C/E/G/H/N/T/V/Y; NS5A: L/M28A/T/V, Q30E/H/R, L31F/I/M/V, H58D or P58S and Y93C/F/H/N/S; NS5B: S282P/R/T, C316H/N/Y, S368T, Y448C/H, S556G/R, D559R.

**Results:**

Variants that require only one transversion in NS3 were found in 4 positions and include F43S, R80K, R155K/G and A156T. The genetic barrier to resistance shows subtypic differences at position 155 of the NS3 gene where a single transition is necessary in subtype 1a. In the NS5A gene, 5 positions where only one nucleotide change can confer resistance were found, such as L31M which requires one transversion in all subtypes, except in 0.28% of 1b sequences; and R30H, generated by a single transition, which was found in 10.25% of the sequences of genotype 1b. Other subtypic differences were observed at position 58, where resistance is less likely in genotype 1a because a transversion is required to create the variant 58S. For the NS5B inhibitors, the genetic barrier at positions conferring resistance was nearly identical in subtypes 1a and 1b, and single transitions or transversions were necessary in 5 positions to generate a drug-resistant variant of HCV. The positions C316Y and S556D required only one transition in all genotypes, Y448H and S556 G/N/R positions required only one transition for up to 98.8% of the sequences analyzed. A single variant in position 448 in genotype 1a is less likely to become the resistance variant 448H because it requires two transversions. Also, in the position 559D a transversion and a transition were necessary to generate the resistance mutant D559H.

**Conclusion:**

Results revealed that in 14 out of 16 positions, conversion to a drug-resistant variant of HCV required only one single nucleotide substitutions threatening direct acting antivirals from all three classes.

## Introduction

First discovered in 1989, hepatitis C virus (HCV) is a major health problem worldwide [1]. The percentage of people who are seropositive for anti-HCV antibodies worldwide is estimated to have increased from 2.3% to 2.8% between 1990 and 2005 [2]. Most patients (80–85%) who become acutely infected cannot clear the virus and progress to chronic infection. Current data states that more than 170 million people are chronically infected by HCV; the outcomes of chronic infection are cirrhosis, portal hypertension, hepatic decompensation, and the development of hepatocellular carcinoma, causing approximately 350,000 deaths per year [3].

HCV contains a positive-sense, single-stranded 9,600 kb RNA genome. A single HCV polyprotein of 3,011 amino acids is translated, and then cleaved by cellular and viral proteases into three structural proteins (core, E1 and E2) and seven non-structural proteins (p7, NS2, NS3, NS4A, NS4B, NS5A, and NS5B) [4]. Additionally, HCV has enormous genetic diversity in infected hosts, existing in blood as a swarm of closely related individual genotypes that may code for subtly distinct phenotypes, known as quasispecies. One of the phenotypes potentially selectable from the quasispecies is drug resistance. HCV diversity derives from an error-prone viral polymerase, rapid replication and natural selection within each host to antibody and cellular immune responses, and now, increasingly, to antiviral drugs [5].

There are two major models explaining the development of drug resistance mutations: the deterministic model and the stochastic model. If the viral effective population size is relatively small, drug resistance mutations might emerge “stochastically” under the selection pressure during treatment with the antivirals, so in this case the genetic barriers of codon changes may affect the development of drug resistance mutations. On the other hand, the deterministic model is based on effective virus population that are large enough to infer that all drug resistance mutations pre-exist and can be seen; this model can be used if there is enough sampling depth (6–8). The model to be used in drug resistance studies depend on the pathogen and population size; there are few studies calculating the effective population size in HCV (6,9).

Factors favoring the emergence of resistant variants include high viral replicative load with prolonged and rapid viral turnover; high intrinsic viral mutation rates; degree of selective drug pressure, which is higher with prolonged or repeated courses of drug therapy, particularly with suboptimal doses; and an antiviral target that can mutate without adversely affecting viral fitness (10).

Advances in our knowledge of the molecular biology of the HCV replication life cycle have led to the development of several molecules that specifically inhibit HCV enzymatic activities that are essential for replication [6, 7]. These compounds are called direct-acting antiviral agents (DAA) and target viral non-structural proteins, including the NS3/4A protease, the NS5B polymerase, and the NS5A protein [8]. Resistance to DAAs is driven by the selection of mutations in the non-structural proteins [9–11]. Each compound or drug family induces a specific mutation profile that is also influenced by the HCV genotype/subtype. Furthermore, each class of DAAs is characterized by a difference in the genetic barrier to resistance; though this general characterization differs for individual agents in the class. Cross-resistance between compounds in the same inhibitor class is of greatest concern for NS3 protease and NS5A inhibitors.

The genetic barrier to resistance, defined as the number of viral mutations required for replication in the presence of drug-selective pressure, is an important factor in HCV treatment. The huge variability between HCV genotypes and subtypes at the nucleotide level could impact the effectiveness of the genetic barrier and therefore, the likelihood of drug resistance development. It has been recognized that despite an identical amino acid at certain positions within the NS3 protease of HCV subtype 1a and 1b the probability/frequency with which a treatment-induced resistance mutation is detected was different between these HCV subtypes. For example, the alteration of arginine at codon position 155 to lysine, which confers resistance to simeprevir and paritaprevir, is 6 times more frequent in genotype 1a than in 1b [12–14]. This is explained, in part, by different nucleotide codons encoding the same amino acid. For instance, two nucleotide changes are required at codon position 155 for generation of R155K in HCV subtype 1b isolates; whereas, in HCV subtype 1a isolates one alteration is sufficient. As a consequence, distinct resistance frequencies are observed in HCV subtype 1a and 1b infected patients after failure to a protease inhibitor based antiviral therapy [15, 16]. Similar differences have been observed also for the generation of other resistance-associated variants (RAVs) in other HCV genes [17, 18].

Beside the number of nucleotide changes required for an amino acid exchange as definition of the genetic barrier to resistance, also the type of exchange seems to be of importance in HCV infection. A model previously described for HIV, HBV, or HCV proposed a score of 1 for transitions and 2.5 for transversions, because it was based on an initial report that addressed the issue of quantifying the genetic barrier for development of drug resistance substitutions between subtypes [19], where it was reported that transitional replacement of a purine by another purine or of a pyrimidine by another pyrimidine are sterically more favorable and therefore occur 2.5-fold more frequently than transversional replacement of a purine by a pyrimidine and vice-versa. Beside this, the HCV RNA is more highly structured than that of HIV or HBV, and more prone to mutations [20]. The HCV NS5B RNA polymerase was shown to favor the generation of nucleotide transitions in comparison to transversions [21]. This may explain that some RAVs are rarely observed at all or generated only after a longer DAA exposure (for example S282T within NS5B leading to resistance to sofusbuvir or L31M within NS5A) causing resistance to ledipasvir [17, 21]. In the case of S282T substitution, the deficit in viral fitness and the low frequencies of transversions over transitions within the diversity of viral quasispecies found by deep-sequencing analyses of HCV samples from treatment-naive patients represent an overall high barrier to the selection of 282T *in vivo* [22, 23] and there is little *in vivo* evidence of drug resistance to nucleo(s/t)ide inhibitors and, when detected, resistant variants with 282T rapidly revert to the wild type as soon as the treatment is interrupted [24, 25].

Currently, there are more than 1,684 complete HCV genome sequences, and 6,567, 6,819 and 1,877 sequences of NS3, NS5A and NS5B, respectively, obtained by Sanger sequencing available on public databanks. Three HCV databases are currently available to provide insights into the basic biology, immunology, and evolution of the virus: the Hepatitis Virus DataBase Server (http://s2as02.genes.nig.ac.jp), the European Hepatitis C Virus database (http://euhcvdb.ibcp.fr) and The Hepatitis C Virus (HCV) Database Project (http://hcv.lanl.gov).

Considering the genetic diversity, the quality and quantity of nucleotide changes and the selection of mutations leading to resistance to DAA’s, the aim of this study was to analyze the impact of genetic variability on the genetic barrier to development of substitutions causing drug resistance to DAAs, quantifying the genetic barrier from the number and type of nucleotide mutations required to impart resistance after analysis of a large number of HCV sequences of all genotypes deposited in European HCV databank.

## Methods

### HCV Database

The sequences analyzed in this study were downloaded in November 2015 from the European Hepatitis C Virus database (https://euhcvdb.ibcp.fr/euHCVdb/). This databank provides key data about the HCV sequences (e.g. genotype, genomic region, viral proteins and their functions, known 3-dimensional structures) and ensures consistency of the annotations, which enables reliable keyword queries. Any user can extract subsets of sequences matching particular criteria or enter their own sequences and analyze them with various bioinformatics programs available on the same server. The euHCVdb is mainly oriented towards protein sequence, structure and function analyses and structural biology of HCV, and is updated every month from a database by an automated process [26].

The search was performed for full-length HCV NS3 protease, NS5A inhibitors and NS5B polymerase sequences segregated by genotype into subtypes 1a, 1b, 2a, 2b and 3a. These subtypes were chosen due to their worldwide prevalence and presence in drug trials, specifically genotype 1 with protease inhibitors and genotype 3 with Polymerase Inhibitor (PI). Reference strains for the three genotypes were obtained (1a: AF009606, 1b: D90208, 2a: D00944, 2b: D10988 and 3a: D17763). Sequences that contain missing data, such as gaps and sequencing errors or were incomplete and sequences from patients previously treated with DAAs were excluded from the analysis. To ensure the quality of the data, sequences were excluded from the analysis if they contained stop codons in the NS5B gene or contained ambiguities consisting of >2 bases per nucleotide position or >2 ambiguities per codon at individual drug resistance-associated position.

### Alignment and edition of the sequences

Sequence alignment was performed with MEGA 6.06 MAC [27] for editing and excluding sequences with missing data (see S1–S15 Files), and for translating the genetic information into amino acids. The resulting protein sequences were then analyzed using BioEdit 7.2.5. software to identify mutations previously associated with resistance [28].

### Genetic barrier calculation

The genetic barrier for each drug resistance substitution was calculated according to a model previously described elsewhere. In summary, transitions (A↔G and C↔T) were assigned a score of 1 and transversions (A↔C, A↔T, G↔C, and G↔T) were assigned a score of 2.5, since transitions have been generally shown to occur for steric reasons on average 2.5 times more frequently than transversions [19, 29, 30]. Briefly, due to the degeneracy of the genetic code, most amino acids associated with drug resistance can be encoded by more than one codon. Therefore, starting from the wild-type codon detected in drug-naive patients, we calculated a numerical score by summing the number of nucleotide transitions and/or transversions required to generate a specific resistance substitution. As a result, we obtained different scores for each pathway of nucleotide mutations required to generate a resistance substitution in response to a given drug. The minimal genetic barrier score for each drug resistance substitution analyzed was considered.

The genetic barrier for each drug resistance substitution within 789 NS3 sequences was investigated: 313 from genotype 1a, 405 from genotype 1b, 18 from genotype 2a, 25 from genotype 2b, and 28 from genotype 3a. We evaluated 708 sequences in the NS5A data set: 274 from genotype 1a, 361 from genotype 1b, 19 from genotype 2a, 26 from genotype 2b, and 28 from genotype 3a. Furthermore, we compiled 536 HCV NS5B sequences: 166 from genotypes 1a, 308 from genotype 1b, 20 from genotype 2a, 24 from genotype 2b, and 18 from genotype 3a. It was included only positions that have been described in previous studies to be associated *in vivo* with treatment failure and/or have been shown *in vitro* phenotypic assays to confer a >2-fold change in replication in comparison to the wildtype reference strain in the presence of the following HCV DAAs: simeprevir (SMV), sofusbuvir (SOF), paritaprevir (PTV), daclatasvir (DCV), ledipasvir (LDV), ombitasvir (OMV), dasabuvir (DSV), grazoprevir (GZR) and elbasvir (EBR). Based on the drug usage recommendations, the following resistance-associated mutations were analysed: NS3: F43/I/L/S/V, Y56H, Q80K/R, R155K/G, A156G/S/T and D168A/C/E/G/H/N/T/V/Y; NS5A: L/M28A/T/V, Q30E/H/R, L31F/I/M/V, P32L, H58D or P58S and Y93C/F/H/N/S; NS5B: C316H/N/Y, S368T, Y448C/H, S556G/R and D559R [31–35].

## Results

Genetic variability among HCV genotypes impacts the calculation of the genetic barrier to development of resistance substitutions. Considering that the available protease inhibitors are less effective against genotypes other than genotype 1 due to natural polymorphisms in their NS3 region, the analyses have taken this into consideration and the discussion focus on the genotype 1 dataset; nevertheless the results of the other genotypes are shown in Table 1. Although some rarely observed NS3 PI resistance variants require transversions or multiple changes, many of the commonly observed changes consist of a single transition to become a resistance mutation (Table 1). Variants that require only one transversion in subtype 1a and 1b include NS3 F43S, R80K, R155K/G and A156T. Subtypic differences were observed at position 155 where a single transition is necessary in subtype 1a but are rare in subtype 1b, where most of the variants require both a transversion and a transition. Some differences in the variants at position 156 are also observed where a transition is necessary to generate the variant 156T but a transversion is necessary to generate 156G or S.

**Table 1:** Codon variability at HCV NS3 positions associated with major drug resistance to IP and its impact on the genetic barrier to drug resistance development in HCV genotypes 1 to 3.

NS3 position	Amino acid	Codon	Frequencies of codon in each genotype					Score
			1a (n = 313)	1b (n = 405)	2a (n = 18)	2b (n = 25)	3a (n = 28)	
43	F	TTC	283 (90.41%)	395 (97.53%)	18 (100%)	23 (92%)	28 (100%)	1.0
		TTT	29 (9.27%)	10 (2.47%)		2 (8%)		1.0
	**S**	**TCC**	**1 (0.32%)**					
80	Q	CAA	151 (48.24%)	53 (13.09%)			3 (10.71%)	2.5
		CAG	16 (5.11%)	325 (80.25%)			25 (89.29%)	2.5
	G	GGC				2 (8%)		4.5
		GGA			4 (22.28%)	5 (20%)		2.0
		GGG			14 (77.78%)	18 (72%)		2.0
	R	AGG	1 (0.32%)					1.0
		CGA	2 (0.64%)					3.5
		CGG	0	1 (0.25%)				3.5
	L	CTA	2 (0.64%)	1 (0.25%)				5.0
		CTG	0	24 (5.93%)				5.0
	**K**	**AAA**	**86 (27.48%)**					
		**AAG**	**55 (17.57%)**	**1 (0.25%)**				
155	R	AGA	49 (15.65%)			24 (96%)	1 (3.57%)	1.0
		AGG	262 (83.71%)	1 (0.25%)		1 (4%)	27 (96.43%)	1.0
		CGA		18 (4.44%)	1 (5.56%)			2.5—155G
		CGG		384 (94.81%)	17 (94.44%)			2.5—155G
	P	CCG		2 (0.49%)				5.0
	**K**	**AAG**	**2 (0.64%)**					
	**G**	**GGC**						
		**GGA**						
		**GGG**						
156	A	GCT		338 (83.46%)		1 (4%)	23 (82.14%)	1.0—156T
		GCC	313 (100%)	66 (16.30%)			5 (17.86%)	2.5—156G/S
		GCA			17 (94.44%)	4 (16%)		1.0—156T
		GCG		1 (0.25%)	1 (5.56%)	20 (80%)		1.0—156T
	**T**	**ACA**						
		**ACG**						
168	D	GAT	12 (3.83%)	28(6.91%)	14 (77.78%)			2.5
		GAC	299 (95.53%)	373 (92.10%)	4 (22.28%)	25 (100%)	28 (100%)	2.5
	Q	CAG					28 (100%)	2.5
	G	GGC	1 (0.32%)					3.5
	A	GCC		1 (0.25%)				5.0
	**E**	**GAA**	**1 (0.32%)**	**4 (0.99%)**				

Amino acids and nucleotides in bold are associated with resistance. Only codons with at least one sequence found in the database are shown, except for the resistance amino acids. The score was calculated considering the minimal change necessary to generate a resistant variant. In cases where more than one variant can be resistant, the nucleotide used as reference is indicated following the score.

The genetic barrier for resistance to NS5A inhibitors appears similar to that for the NS3 PIs, with the majority of variants requiring a single transition in two of the five positions analyzed (Table 2). Exceptions include the NS5A L31M variant, which requires at least one transversion in all subtypes (except in 0.28% of the 1b sequences). Also, in 10.25% of the sequences of genotype 1b, at position 30 a single transition is necessary to generate the mutation R30H which causes resistance to DCV, LDV and OMV. Other subtypic differences were observed at position 58 where genotype 1a seems to confer some protection against development of resistance, because a transversion is required to create the variant 58S, which leads to resistance to DCV, compared to single transitions in other subtypes.

**Table 2:** Codon variability at HCV NS5A positions associated with major drug resistance to NS5A inhibitors and its impact on the genetic barrier to drug resistance development in HCV genotypes 1 to 3.

NS5A position	Amino acid	Codon	Frequencies of codon in each genotype					Score
			1a (n = 274)	1b (n = 361)	2a (n = 19)	2b (n = 26)	3a (n = 28)	
28	M	ATG	259 (94.53%)	10 (2.77%)			27 (96.48%)	1.0
	V	GTG	12 (4.38%)	2 (0.55%)				2.0
	I	ATT	1 (0.36%)					1.0
		ATA					1 (3.57%)	1.0
	F	TTT			3 (15.79%)			3.5
		TTC			16 (84.21%)			3.5
	L	TTG		10 (2.77%)				5.0
		CTT		1 (0.28%)		2 (7.69%)		3.5
		CTC				24 (92.31%)		3.5
		CTA		6 (1.66%)				3.5
		CTG		332 (91.97%)				3.5
	**T**	**ACG**	**2 (0.73%)**					
30	Q	CAA	260 (94.89%)	3 (0.83%)				2.5
		CAG	9 (3.28%)	20 (5.54%)				2.5
	R	AGG		1 (0.28%)	1 (5.26%)			6.0
		CGT		1 (0.28%)				1.0
		CGC		1 (0.28%)				3.5
		CGA	1 (0.36%)	37 (10.25%)				3.5
		CGG		290 (80.33%)				3.5
	K	AAA		4 (1.11%)	4 (21.05%)	24 (92.31%)		5.0
		AAG			14 (73.68%)	2 (7.69%)		5.0
	L	TTG					1 (3.57%)	6.0
		CTG		1 (0.28%)				5.0
	M	ATG		1 (0.28%)				6.0
	A	GCA					2 (7.14%)	7.5
		GCG					25 (89.29%)	7.5
	**H**	**CAT**	**1 (0.36%)**					
		**CAC**	**3 (1.09%)**					
31	L	TTA		184 (50.97%)				3.5
		TTG	19 (6.93%)	104 (28.81%)				2.5
		CTT	5 (1.82%)	1 (0.28%)				5.0
		CTC	4 (1.46%)				28 (100%)	5.0
		CTA	1 (0.36%)	28 (7.76%)				3.5
		CTG	242 (88.32%)	30 (8.31%)	3 (15.79%)	6 (23.08%)		2.5
	I	ATA		1 (0.28%)				1.0
	**M**	**ATG**	**3 (1.09%)**	**12 (3.32%)**	**16 (84.21%)**	**20 (76.92%)**		
58	H	CAT	7 (2.55%)					2.5
		CAC	253 (92.34%)					2.5
	C	TGT	4 (1.46%)					3.5
	P	CCT	3 (1.09%)	2 (0.55%)	16 (84.21%)		27 (96.48%)	1.0—58S
		CCC	3 (1.09%)	7 (1.94%)	2 (10.53%)			5.0
		CCA		298 (82.55%)		22(84.62%)		1.0—58S
		CCG		29 (8.03%)		3 (11.54%)		1.0—58S
	R	CGT					1 (3.57%)	3.5
		CGC	1 (0.36%)					3.5
	Y	TAT	1 (0.36%)					2.5
	T	ACA		5 (1.39%)				2.5—58S
		ACG		1 (0.28%)				2.5—58S
	Q	CAA		2 (0.55%)				5.0
	A	GCA		1 (0.28%)				2.5—58S
	L	CTA		3 (0.83%)				2.0—58S
	**S**	**AGC**						
		**AGT**						
		**TCT**		**1 (0.28%)**	**1 (5.26%)**	**1 (3.85%)**		
		**TCA**		**12 (3.32%)**				
93	Y	TAT	17 (6.20%)	4 (1.11%)	2 (10.63%)		3 (10.71%)	1.0
		TAC	254 (92.70%)	338 (93.63%)	17 (89.47%)	26 (100%)	25 (89.29%)	1.0
	R	CGT		3 (0.83%)				1.0
	**C**	**TGT**		**1 (0.28%)**				
		**TGC**	**1 (0.36%)**					
	**H**	**CAT**		**15 (4,25%)**				
		**CAC**	**2 (0.73%)**					

Amino acids and nucleotides in bold are associated with resistance. Only codons with at least one sequence found in the database are shown, except for the resistance amino acids. The score was calculated considering the minimal change necessary to generate a resistant variant. In cases where more than one variant can be resistant, the nucleotide used as reference is indicated following the score.

The genetic barrier differs for the various NS5B inhibitor classes (nucleo(s/t)ide, Palm site, Thumb site, Finger-loop) (Table 3). For the NS5B inhibitors, while subtypic differences in activity are known, the genetic barrier at positions conferring resistance were nearly identical in subtypes 1a and 1b, and the majority were single transitions or transversions. The positions C316Y and S556D where palm inhibitors NS5B take action required only one transition in all genotypes, Y448H and S556 G/N/R positions required only one transition for up to 98.8% of sequences analyzed. A single variant with a glutamic acid replacing the tyrosine in position 448 can confer some protection in genotype 1a because it then necessitates two transvertions to become the resistance variant 448H. Also, in the position 559D a transvertion and a transition were necessary to become a resistance mutant D559H.

**Table 3:** Codon variability at HCV NS5B positions associated with major drug resistance to NS5B inhibitors and its impact on the genetic barrier to drug resistance development in HCV genotypes 1 to 3.

NS5B position	Amino acid	Codon	Frequencies of codon in each genotype					Score
			1a (n = 166)	1b (n = 308)	2a (n = 20)	2b (n = 24)	3a (n = 18)	
282	S	AGC	164 (98.8%)	293 (95.13%)	19 (95%)	24 (100%)	3 (16.67%)	2.5
		AGT	1 (0.66%)	15 (4.87%)	1 (5%)		14 (77.78%)	2.5
	P	CCG					1 (5.56%)	2.5
	R	AGG	1 (0.66%)					2.5
	**T**	**ACA**						
		**ACG**						
316	C	TGT	152 (91.57%)	7 (2.27%)		24 (100%)	1 (5.56%)	1.0
		TGC	14 (8.43%)	185 (60.06%)	20 (100%)		17 (94.44%)	1.0
	R	CGC		1 (0.32%)				2.0
	N	AAT		3 (0.97%)				2.5
		AAC		111 (36.04%)				2.5
	**Y**	**TAT**						
		**TAC**		**1 (0.32%)**				
368	S	TCT	40 (24.10%)	5 (1.62%)			4 (22.22%)	2.5
		TCC	123 (74.10%)	298 (96.75%)			12 (66.67%)	2.5
		TCA		3 (0.97%)	19 (95%)	24 (100%)	2 (11.11%)	2.5
		TCG	3 (1.81%)	1 (0.32%)	1 (5%)			2.5
	P	CCC		1 (0.32%)				2.5
	**T**	**ACA**						
		**ACG**						
448	Y	TAT	2 (1.20%)	18 (5.84%)	2 (10%)	9 (37.50%)		1.0
		TAC	162 (97.59%)	290 (94.16%)	18 (90%)	15 (62.50%)	18 (100%)	1.0
	E	GAA	1 (0.66%)					5.0
		GAG	1 (0.66%)					5.0
	**H**	**CAT**						
		**CAC**						
556	S	AGT		270 (87.66%)				1.0
		AGC	166 (100%)	5 (1.62%)				1.0
	D	GAC		3 (0.97%)				1.0
	**G**	**GGC**		**24 (7.79%)**	**20 (100%)**	**24 (100%)**	**18 (100%)**	
	**N**	**AAC**		**6 (1.95%)**				
559	D	GAT	1 (0.66%)					3.5
		GAC	165 (99.34%)	307 (99.68%)	19 (95%)	24 (100%)	18 (100%)	3.5
	N	AAC		1 (0.32%)				3.5
	**H**	**CAT**						
		**CAC**			**1 (5%)**			

Amino acids and nucleotides in bold are associated with resistance. Only codons with at least one sequence found in the database are shown, except for the resistance amino acids. The score was calculated considering the minimal change necessary to generate a resistant variant.

Regardless of HCV genotype the analysis revealed that in 14 of 16 positions conversion to a drug-resistant variant required only single nucleotide substitutions (Table 4). That is, one transition with a genetic barrier score of 1 (F43S, Q80R, R155K or G, A156T in NS3 gene; H or P58S, Y93H in NS5A; C316Y and S556N in NS5B), or one transversion with a genetic barrier score of 2.5 (Q80K, A156S, D156E in NS3 gene; and S368T in NS5B gene).

**Table 4:** Resistance level to DDA at HCV NS3, NS5A and NS5B positions and level of genetic barrier.

Gene	Drug	Mutation	Codon	Frequencies in each genotype					Resistance level
				1a	1b	2a	2b	3a	
*NS3*	Simeprevir	Q80K	CAA^§^	151 (48.24%)	53 (13.09%)			3 (10.71%)	low
			CAG^§^	16 (5.11%)	325 (80.25%)			25 (89.29%)	
		R155K	AGA^#^	49 (15.65%)					high
			AGG^#^	262 (83.71%)	1 (0.25%)				
			CGA^#§^		18 (4.44%)				
			CGG^#§^		384 (94.81%)				
		D168E	GAT^§^	12 (3.83%)	28(6.91%)	14 (77.78%)			low-intermediate
			GAC^§^	299 (95.53%)	373 (92.10%)	4 (22.28%)	25 (100%)		
	Paritaprevir	R155K	AGA^#^	49 (15.65%)					high
			AGG^#^	262 (83.71%)	1 (0.25%)				
			CGA^#§^		18 (4.44%)				
			CGG^#§^		384 (94.81%)				
		D168E	GAT^§^	12 (3.83%)	28(6.91%)	14 (77.78%)			low-intermediate
			GAC^§^	299 (95.53%)	373 (92.10%)	4 (22.28%)	25 (100%)		
	Grazoprevir	A156T	GCT^#^		338 (83.46%)		1 (4%)	23 (82.14%)	low-intermediate
			GCC^§^	313 (100%)	66 (16.30%)			5 (17.86%)	
			GCA^#^			17 (94.44%)	4 (16%)		
			GCG^#^		1 (0.25%)	1 (5.56%)	20 (80%)		
		D168E	GAT^§^	12 (3.83%)	28(6.91%)	14 (77.78%)			low-intermediate
			GAC^§^	299 (95.53%)	373 (92.10%)	4 (22.28%)	25 (100%)		
*NS5A*	Ledipasvir	L31M	TTA^#§^		184 (50.97%)				low-high
			TTG^§^	19 (6.93%)	104 (28.81%)				
			CTT^§§^	5 (1.82%)	1 (0.28%)				
			CTC^§§^	4 (1.46%)				28 (100%)	
			CTA^#§^	1 (0.36%)	28 (7.76%)				
			CTG^§^	242 (88.32%)	30 (8.31%)				
		Y93H	TAT^#^	17 (6.20%)	4 (1.11%)	2 (10.63%)		3 (10.71%)	high
			TAC^#^	254 (92.70%)					
	Daclatasvir	M28T	ATG^#^	259 (94.53%)	10 (2.77%)			27 (96.48%)	intermediate
		Q30H	CAA^§^	260 (94.89%)	3 (0.83%)				low-high
			CAG^§^	9 (3.28%)	20 (5.54%)				
		Y93H	TAT^#^	17 (6.20%)	4 (1.11%)	2 (10.63%)		3 (10.71%)	high
			TAC^#^	254 (92.70%)					
	Ombitasvir	M28T	ATG^#^	259 (94.53%)	10 (2.77%)			27 (96.48%)	intermediate
		Y93H	TAT^#^	17 (6.20%)	4 (1.11%)	2 (10.63%)		3 (10.71%)	high
			TAC^#^	254 (92.70%)					
	Elbasvir	M28T	ATG^#^	259 (94.53%)	10 (2.77%)			27 (96.48%)	intermediate
		Q30H	CAA^§^	260 (94.89%)	3 (0.83%)				low-high
			CAG^§^	9 (3.28%)	20 (5.54%)				
		L31M	TTA^#§^		184 (50.97%)				low-high
			TTG^§^	19 (6.93%)	104 (28.81%)				
			CTT^§§^	5 (1.82%)	1 (0.28%)				
			CTC^§§^	4 (1.46%)				28 (100%)	
			CTA^#§^	1 (0.36%)	28 (7.76%)				
			CTG^§^	242 (88.32%)	30 (8.31%)				
		Y93H	TAT^#^	17 (6.20%)	4 (1.11%)	2 (10.63%)		3 (10.71%)	high
			TAC^#^	254 (92.70%)					
*NS5B*	Sofusbuvir	S282T	AGC^§^	164 (98.8%)	293 (95.13%)	19 (95%)	24 (100%)	3 (16.67%)	high
			AGT^§^	1 (0.66%)	15 (4.87%)	1 (5%)		14 (77.78%)	
			TCT						
			TCC						
			TCA						
			TCG						
	Dasabuvir	C316Y	TGT^#^	152 (91.57%)	7 (2.27%)	20 (100%)	24 (100%)	1 (5.56%)	high
			TGC^#^	14 (8.43%)	185 (60.06%)			17 (94.44%)	
		C316N	AAT^#§^	152 (91.57%)	7 (2.27%)	20 (100%)	24 (100%)	1 (5.56%)	low
			AAC^#§^	14 (8.43%)	185 (60.06%)			17 (94.44%)	
		S556G	AGT^#^		270 (87.66%)				intermediate
			AGC^#^	166 (100%)	5 (1.62%)				
		S556N	AGT^#^		270 (87.66%)				
			AGC^#^	166 (100%)	5 (1.62%)				

^#^one transition: score 1.0;

^§^one transversion: score 2.5;

^#§^one transition + one transversion: score 3.5;

^§§^two transversions: score 5.0

## Discussion

Besides the genetic variability and natural presence of drug resistance substitutions in selected genotypes prior to treatment, another factor that can be associated with probability of success of a DAA-based regimen is the genetic barrier for the development of resistance. This can be broadly defined as the number and type of nucleotide mutations required for the generation of a specific resistance substitution, starting from the wild-type genetic background of the virus [22]. Previous studies have shown that genetic variability among HIV, HBV, or HCV genotypes can in some cases facilitate the development of specific resistance variants [19, 22, 23, 29, 30, 36]. For instance, it has recently been proposed that the high degree of HCV genetic variability makes HCV genotypes, and even subtypes, differently prone to the development of PI resistance substitutions, with important clinical implications for tailoring individualized and appropriate regimens [29]. These findings support that genetic barrier is higher for genotype 1b than to 1a in the NS3 gene.

These results are consistent with available HCV experimental and clinical treatment observations [37]. Within NS3, many sequences had shown the Q80K mutation (44.7% for genotype 1a, 0.25% for genotype 1b), which can cause resistance to SMV, PTV and ASV but how Q80K alters GZR susceptibility in cell culture has not been reported to our knowledge (Tables 1 and 4). The R155K mutation was rarely observed in subtype 1b viruses, where two changes (one transition and one transversion) are required, while subtype 1a needs only one transition. A similar profile has also been observed for other PIs such as SMV, FDV, ASV and PTV/r, to which subtype 1a is more prone to acquire RAVs (Table 4).

Regarding NS5A RAVs, Y93H in genotype 1b was the most commonly variant identified (4.25%), followed by L31M (3.40%), whereas other NS5A RAVs occurred at low frequencies. Furthermore, the overall number of sequences with some NS5A RAVs was higher compared to other genes but also the genetic barrier in this class is higher (requiring changing in more than one nucleotide). Differences in the level of resistance depending on the HCV subtype were seen in all position except in 93. The genetic barrier to DAA’s acting in NS5B gene was lower than for NS5A, with exception of positions 448 and 559 where at least two mutations were shown to be necessary to generate a RAV. Similarly, two substitutions, 316Y and 448H, with low genetic barriers were shown to strongly reduce HCV susceptibility to DAA’s; accordingly, the 316Y variant was described to confer resistance to DSV [38, 39]. Finally, the major resistance variant S282T, selected *in vitro* by SOF, requires only a single G-to-C transversion (score 2.5) and is rarely, if ever, seen in clinical isolates [11, 22], because it alters the conformation of the enzyme catalytic site [40] and severely compromises viral fitness among different HCV genotypes [41, 42].

All RAVs found in this study were identified in näıve-treatment patients. However, considering that the present analysis is based on a databank, this findings are only correlative rather than conclusive and the clinical relevance of this data is yet to be confirmed by additional longitudinal follow-up studies with DAAs involving patients infected with distinct HCV genotypes. The analysis of genetic variation suggests that sequence variation is wide and therefore some patients likely have a lower barrier of resistance than others; on the other hand, clinical data currently available argue that current DAA regimens have sufficient multiplicity of action and duration, and that treatment failure is rare.

Given that all oral HCV DAA therapies are associated with high costs, resistance testing at baseline, which is also a significant cost, may nevertheless be worthwhile to identify the best DAA treatment option for each patient depending on the HCV genotype and preexisting polymorphisms in the NS3 and NS5A/B [37]. However, which frequencies of pre-existing RAVs within the HCV quasispecies and which level of resistance of pre-existing RAVs may contribute to treatment failure is not completely clarified. The presence in DAA-naive patients of natural polymorphisms at resistance positions in selected genotypes, together with a broad low genetic barrier for the development of resistance represents indeed an important issue in the global approach for the management and treatment of HCV-related disease.

## Supporting Information

S1 File euhcvdb-NS3-1a. (FAS)

S2 File euhcvdb-NS3-1b. (FAS)

S3 File euhcvdb-NS3-2a. (FAS)

S4 File euhcvdb-NS3-1b. (FAS)

S5 File euhcvdb-NS3-3a. (FAS)

S6 File euhcvdb-NS5a-1a. (FAS)

S7 File euhcvdb-NS5a-1b. (FAS)

S8 File euhcvdb-NS5a-2a. (FAS)

S9 File euhcvdb-NS5a-2b. (FAS)

S10 File euhcvdb-NS5a-3a. (FAS)

S11 File euhcvdb-NS5b-1a. (FAS)

S12 File euhcvdb-NS5b-1b. (FAS)

S13 File euhcvdb-NS5b-2a. (FAS)

S14 File euhcvdb-NS5b-2b. (FAS)

S15 File euhcvdb-NS5b-3a. (FAS)
